# Morphology and surface analyses for CH_3_NH_3_PbI_3_ perovskite thin films treated with versatile solvent–antisolvent vapors

**DOI:** 10.1039/d1ra02645c

**Published:** 2021-05-17

**Authors:** Nasir Awol, Chernet Amente, Gaurav Verma, Jung Yong Kim

**Affiliations:** School of Materials Science and Engineering, Jimma Institute of Technology, Jimma University P. O. Box 378 Jimma Ethiopia jungyong.kim@ju.edu.et; Dr Shanti Swarup Bhatnagar University Institute of Chemical Engineering and Technology, Panjab University Chandigarh 160014 India gauravverma@pu.ac.in; Department of Physics, College of Computational and Natural Science, Addis Ababa University P. O. Box 1176 Addis Ababa Ethiopia; Centre for Nanoscience & Nanotechnology, University Institute for Emerging Areas in Science and Technology, Panjab University Chandigarh 160014 India; School of Chemical Engineering, Jimma Institute of Technology, Jimma University P. O. Box 378 Jimma Ethiopia

## Abstract

Organometal halide perovskite (CH_3_NH_3_PbI_3_) semiconductors have been promising candidates as a photoactive layer for photovoltaics. Especially for high performance devices, the crystal structure and morphology of this perovskite layer should be optimized. In this experiment, by employing solvent–antisolvent vapor techniques during a modified sequential deposition of PbI_2_–CH_3_NH_3_I layers, the morphology engineering was carried out as a function of antisolvent species such as: chloroform, chlorobenzene, dichlorobenzene, toluene, and diethyl ether. Then, the optical, morphological, structural, and surface properties were characterized. When dimethyl sulfoxide (DMSO, solvent) and diethyl ether (antisolvent) vapors were employed, the CH_3_NH_3_PbI_3_ layer exhibited relatively desirable crystal structures and morphologies, resulting in an optical bandgap (*E*_g_) of 1.61 eV, crystallite size (*t*) of 89.5 nm, and high photoluminescence (PL) intensity. Finally, the stability of perovskite films toward water was found to be dependent on the morphologies with defects such as grain boundaries, which was evaluated through contact angle measurement.

## Abstract 2

Organometal halide perovskite (CH_3_NH_3_PbI_3_) semiconductors have been promising candidates as a photoactive layer for photovoltaics.

## Graphical abstract

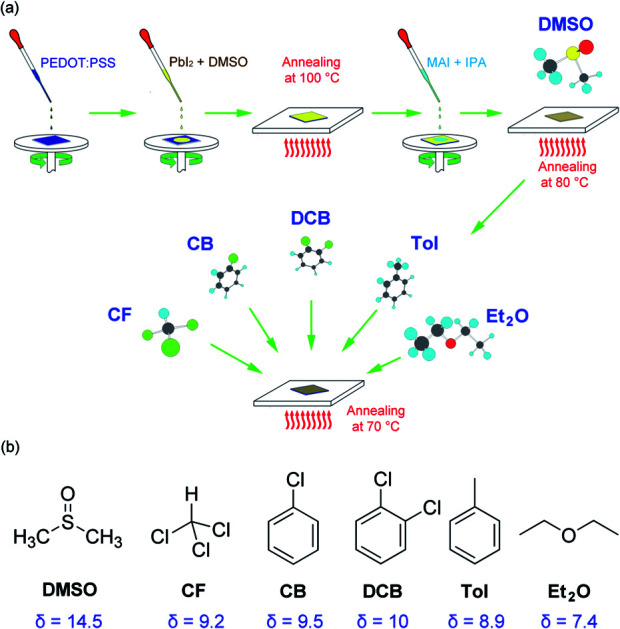

## 1. Introduction

Organometal halide perovskite solar cells (PSCs) have received tremendous interest for a next-generation photovoltaic (PV) technology.^1–5^ Perovskite can be designated by a common formula known as ABX_3_ where ‘A’ is a large organic cation [CH_3_NH_3_ or HC (NH_2_)_2_], ‘B’ is a metal cation (Pb, Sn), and ‘X’ is a halide (Cl, Br, I). The perovskite material is a light-harvesting component of the PSCs, and is able to offer many desirable characteristics such as low-temperature solution processability,^6,7^ high absorption coefficient,^8^ long carrier diffusion length,^9^ high charge carrier mobility,^10^ and adjustable direct bandgap with suitable alternative metals, halogens, and organic cations.^11–14^ These characteristics can be further modified by using additives,^15–17^ compositional adjustments,^18,19^ and solvent–antisolvent extraction approaches.^20^ Hence, the PSCs have been a promising candidate for commercialization in the current PV industries.

In general, the PV performance of PSCs relies on the morphologies of the perovskite thin film because the structural characteristics of a photoactive layer decide PV performances of devices.^21–32^ For example, if there is a trap site (*e.g.*, surface defect and grain boundary) in a perovskite layer, it acts as carrier recombination sites,^33^ resulting in a reduced performance of devices. Thus, the morphology and crystallinity of the perovskite thin film should be very important for fabricating high-efficiency PV devices.^34^

To date, numerous approaches have been developed to obtain a high quality and defect-minimized perovskite thin film.^35,36^ For example, thermal annealing of a perovskite film at 85–120 °C has been employed.^37^ Furthermore, low-temperature antisolvent assisted fabrication of devices are one of the useful techniques for obtaining a film with desired morphologies.^18,19^ Importantly, it is notable that the additive and antisolvent strategies are both significantly promising in improving the performance of PSCs.^38–49^ Moreover, the dipping time,^50^ precursor's type and concentration,^51^ spin-speed,^52^ solvent types,^53,54^ and temperature are important processing factors for optimizing a perovskite layer. In the sequential deposition of the PbI_2_ and CH_3_NH_3_I (MAI) layers, the MAI's intercalation into the PbI_2_ layer is critically important to obtain a high quality perovskite without any unreacted precursor material. If there is an incomplete conversion of PbI_2_–MAI into a perovskite, it may be a problem for device performances.^55^ However, for improving the stability of PSCs, there are researchers who used a PbI_2_ interfacial nanolayer in their device configuration.^56–60^

In this work, we employed a modified sequential deposition method for fabricating organometal halide perovskite thin films. For this purpose, the solvent–antisolvent vapor techniques were adopted as a method of morphology engineering. The five anti-solvents such as chloroform (CF), chlorobenzene (CB), 1,2-dichlorobenzene (DCB), toluene (Tol), and diethyl ether (Et_2_O) were tested, which may act as an extractor of a solvent, dimethyl sulfoxide (DMSO). Then the properties of CH_3_NH_3_PbI_3_ thin films were investigated as a function of antisolvent species, which may include UV-vis light absorption, micro-/nano-structural morphologies, crystal structures, photoluminescence (PL) emission, and surface analysis through the water contact-angle measurements. In this study, it was observed that when a perovskite layer is well crystallized, the surface polarity of perovskite films remains a longer time, *i.e.*, an enhanced stability toward water or its vapor.

## 2. Experimental section

### 2.1. Materials and methods

In all synthesis methods, analytical grade high purity reagents were used. All solvents and antisolvents were purchased from Fine Chemicals Ltd. Indium tin oxide/fluorine-doped tin oxide (ITO/FTO) coated glass substrates were purchased from TECHINSTRO Chemicals Ltd. PbI_2_ precursors were purchased from Tokyo chemical industries (TCI) and synthesized using a hydrothermal method.^27^ CH_3_NH_3_I (MAI) was synthesized by reacting methylamine (aqueous, 40 wt%) and hydroiodic acid (aqueous, 57 wt%) in an ice bath for 2 h with stirring. Then the solvent was evaporated using a rotary evaporator and the precipitate was collected and washed using Et_2_O three times and dried at 60 °C for 24 h in a vacuum oven. The resulting product, MAI, was used without further purification. To obtain a CH_3_NH_3_PbI_3_ precursor, the synthesized PbI_2_ and MAI were deposited on the top of poly(3,4-ethylenedioxythiophene):polystyrene sulfonate (PEDOT:PSS)-coated substrate using a modified sequential deposition technique: (a) PbI_2_/DMSO deposition, (b) MAI/IPA deposition at a 1 : 1 mole ratio, and (c) solvent–antisolvent exposure, in which the solvent is DMSO, and the antisolvents are CF, CB, DCB, Tol, and Et_2_O.

### 2.2. Thin-film preparation

To study the effect of a solvent, DMSO was used to prepare 1 M of PbI_2_ (461.78 mg ml^−1^ of DMSO) solution and annealed at 80 °C for 12 hours. ITO coated glass substrates were used to deposit the samples and sequentially washed with detergent, DI water, and ethanol in an ultrasonic bath. A hole transporting material PEDOT:PSS was deposited on the top of the ITO glass substrate. Then, the PbI_2_ precursor solution was filtered by a 2 μm sterile polytetrafluoroethylene (PTFE) membrane filter. PbI_2_/DMSO was spin-coated on the top of the PEDOT:PSS layer and heated at a temperature of 100 °C. Then, the as-synthesized MAI in isopropyl alcohol (IPA) solution was spin-coated on the top of the PbI_2_ layer. After deposition of the MAI, the thin film was exposed to a DMSO vapor at 80 °C for 10 minutes, and then the crystallizable perovskite layer was exposed to different antisolvent vapor at 70 °C for 10 minutes. The optical, structural, morphological, and surface properties of organometal halide perovskite thin films were then investigated, accordingly.

### 2.3 Characterization

UV-visible spectra measurements were taken on thin films using Shimadzu UV-2600-Series, diffused reflectance spectrophotometer double-beam source at a range of 350–800 nm fitted with deuterium and halogen lamps as sources. Transmission and reflection modes were recorded simultaneously. ITO glasses have been used as a background reference for thin films. The X-ray diffraction (XRD) patterns of the as-prepared perovskite samples have been characterized by Philips X'pert PRO-240 mm diffractometer provided with an integrated germanium detector Cu-Kα, radiation source at *λ* = 1.54060 Å operating at an applied voltage of 45 kV with a current intensity of 40 mA. The equatorial scans in the continuous mode were taken from 2*θ* = 4° to 80° at a step of 0.017° with a scan step time of 24.4 seconds. To probe the electron transition behavior of samples photoluminescence (PL) spectroscopy was utilized by F-7000 fluorescence spectrophotometer, Hitachi, Japan, using a xenon lamp at a wavelength of 532 nm with an emission wavelength starting from 600 nm to 850 nm at a scan speed of 1200 nm min^−1^. The morphologies of perovskite films were investigated by field-emission scanning electron microscopy (FE-SEM; Hitachi, Japan, SU8000 Series) at an accelerating voltage of 5.0 kV. Energy-dispersive X-ray spectroscopy (EDX) investigation has been performed to probe the elemental distribution variations present in the thin film. Mapping analysis has been done to obtain elemental maps in the range of nanoscale. Contact Angle Goniometer (KRUSS GmbH, DSA25; Germany) was used to record and analyze the effect of antisolvents on the surface energies of perovskite thin films. To determine contact angle, the edge detection of the water droplet was fitted by a polynomial fitting approach. Measurements were taken in time intervals of 40 ms over a period of 30 seconds.

## 3. Results and discussion

**Fig. 1:**
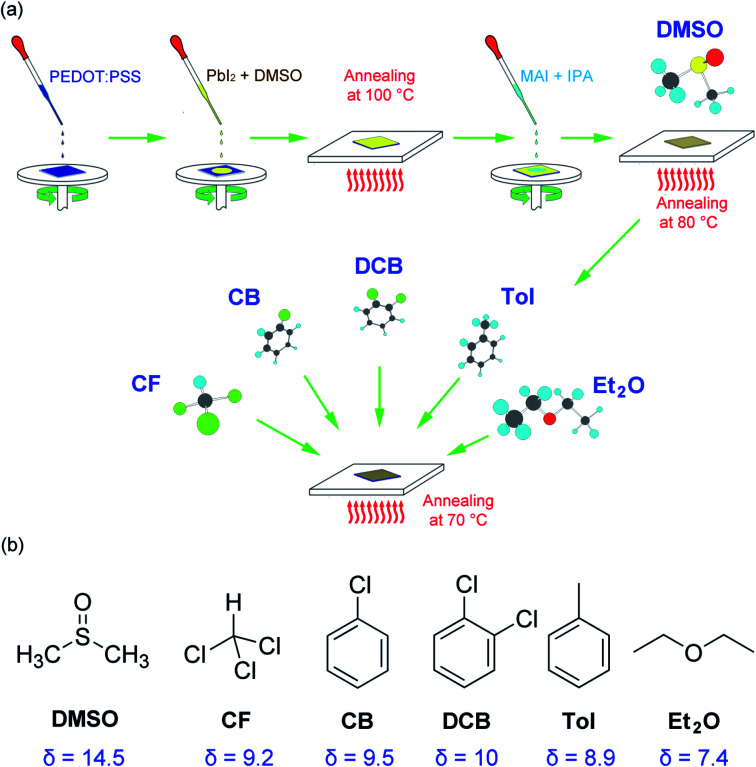
(a) A modified sequential deposition technique for perovskite film, for which a solvent–antisolvent vapor-exposure technique was employed for morphology engineering. (b) Chemical structures of solvent and antisolvents for organometal halide perovskites. Here *δ* is the solubility parameter with a dimension of (cal cm^−3^)^1/2^.

Fig. 1(a) shows a modified sequential deposition of the perovskite layer. For the first step, PbI_2_ is deposited on the top of PEDOT:PSS/ITO substrate, then, the MAI is spin coated on the top of PbI_2_/PEDOT:PSS/ITO. Finally, the solvent (DMSO) and antisolvent (CF, CB, DCB, Tol, Et_2_O) vapors are sequentially exposed to the perovskite layer. The properties of solvent and antisolvents were summarized in Table 1. Here, the solubility parameter (*δ*) with the dimension of (cal cm^−3^)^1/2^ is in the order of 14.5 (DMSO) > 10 (DCB) > 9.5 (CB) > 9.2 (CF) > 8.9 (Tol) > 7.4 (Et_2_O), indicating that DMSO is the most polar, whereas Et_2_O is the most nonpolar. Furthermore, the boiling point (bp) is in the order of 189 °C (DMSO) > 180 °C (DCB) > 131 °C (CB) > 111 °C (Tol) > 61 °C (CF) > 35 °C (Et_2_O), displaying Et_2_O is the most volatile.

**Table 1:** Chemical formula, solubility parameter,^64^ molecular weight, and density and boiling point (bp) of solvent or antisolvent in this study

Molecule	Chemical formula	*δ* a (cal cm^−3^)^1/2^	*δ* MPa^1/2^	MW (g mol^−1^)	*ρ* (g cm^−3^)	bp (°C)
DMSO	C_2_H_6_OS	14.5	29.71	78.13	1.10	189
CF	CHCl_3_	9.2	18.85	119.38	1.49	61
CB	C_6_H_5_Cl	9.5	19.47	112.56	1.11	131
DCB	C_4_H_4_Cl_2_	10.0	20.49	147.01	1.30	180
Tol	C_7_H_8_	8.9	18.24	92.14	0.87	111
Et_2_O	C_4_H_10_O	7.4	15.16	74.12	0.71	35

a In the text, the dimension of (cal cm^−3^)^1/2^ was used for solubility parameter.

For solvent–antisolvent vapor engineering, the solvent/antisolvent should be miscible, whereas the perovskite/antisolvent immiscible. During the film-formation process, if the number of nucleation cites is reduced, the crystal and grain size of perovskite may increase, resulting in a high quality film with small grain boundaries. For this purpose, the solvent DMSO molecules should be quickly extracted from the wet DMSO/perovskite film by help of antisolvent.^61,62^ Furthermore, it is notable that although perovskite is hygroscopic and hydrophilic, the measured water-contact angle was reported to be very high (*i.e.*, significantly hydrophobic). This paradox was solved by recognizing that the hydrophobic PbI_2_ is formed at the interface of water and CH_3_NH_3_PbI_3_.^63^ In other words, the measured water contact angle is not for CH_3_NH_3_PbI_3_, but for PbI_2_ (*i.e.*, the result of a perovskite degradation). Here, of course, the contact angle data may include the effect of the morphologies of a film including grain boundaries.

Fig. 1(b) shows the chemical structure of solvent and antisolvents. Here the solubility parameter (*δ*) is equal to a square root of cohesive energy density (CED), *i.e.*, 
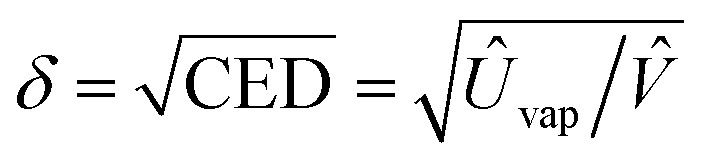
, where *Û*_vap_ is the molar heat of vaporization, and *V̂* is the molar volume.^64^ Furthermore, two small organic molecules (here, solvent and antisolvent) are expected to be miscible because of a large entropic gain, although there is an enthalpic cost from the apparent dissimilarity in solubility parameters. Hence, Δ*G*_mix_ = Δ*H*_mix_ − *T*Δ*S*_mix_ < 0, in which Δ*G*_mix_, Δ*H*_mix_, andΔ*S*_mix_ denote the Gibbs free energy, enthalpy, and entropy of mixing, respectively, and *T* is temperature. On the other hand, for the intermolecular interactions between antisolvent and perovskite, the relation should be Δ*G*_mix_ > 0, facilitating a wet perovskite film to undergo a drying process.

**Fig. 2:**
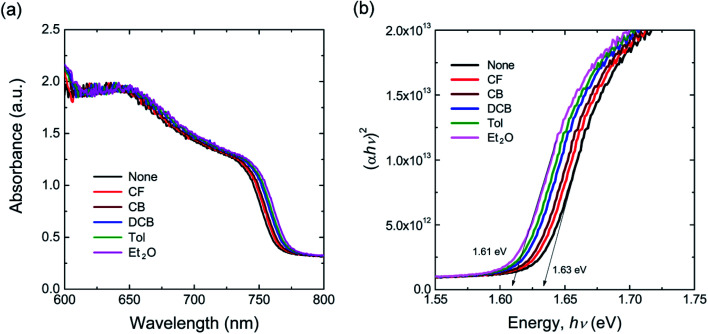
(a) UV-vis absorption spectra of the perovskite thin films as a function of nonsolvent species. (b) The plot (*αhν*)^2^*vs. hν*: optical bandgaps of each perovskite film.

Fig. 2(a) shows the UV-vis absorption spectra of perovskite films as a function of antisolvent species. As shown in Fig. 2(a), although the overall shape of absorption is similar, the absorption edge, *i.e.*, the optical bandgap (*E*_g_), is a little bit different due to a non-identical ordering state of a film. Here, the absorption data was replot using the Tauc model,^65^1
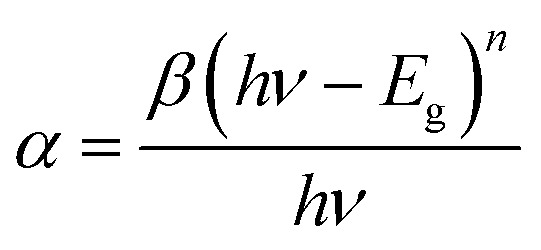
where *α* is the absorption coefficient, *β* is a constant (disorder parameter), *h* is Plank's constant and *ν* is the frequency of light. The value ‘*n*’ is 1/2 for a direct bandgap semiconductor and 2 for an indirect bandgap.^66^ Hence, *n* = 1/2 can be used because CH_3_NH_3_PbI_3_ is included in the former.

As shown in Fig. 2(b), the plot (*αhν*)^2^*vs. hν*, results in the optical bandgap of ∼1.61–1.63 eV. As an example, the absorption edge is 770.19 nm (*E*_g_ = 1.61 eV) for Et_2_O vapor condition, whereas it is 760.74 nm (*E*_g_ = 1.63 eV) for ‘None’ condition, *i.e.*, the perovskite sample was not exposed to any solvent/antisolvent vapor. Here, the small bandgap indicates that the perovskite semiconductor has a well-organized structure, as observed in other stereoregular polymer semiconductors through red-shift in the absorption spectra.^67–69^ Note that, if the perovskite becomes a single crystalline wafer, the bandgap was reported to be much smaller like 1.36 eV, corresponding to the light absorption onset at 910 nm.^70^ This trend indicates that the allowed energy states of an electron increase with reducing defect densities in the crystalline lattice forming a periodic potential. In other words, the energy band increases and the bandgap decreases if the quality of perovskite films is improved. Furthermore, if there are any defects in perovskite, the typical trap energies are known to be shallow because of its defect-tolerance property.^71–73^ Hence, based on the optical data, the ordering of perovskite materials is in the order of: Et_2_O > Tol > DCB > CB > CF > ‘None’. Interestingly, if there is no solvent–antisolvent vapor treatment, the perovskite sample exhibits the smallest optical absorption, indicating that the vapor treatment is a useful technique for organizing the perovskite films.

**Fig. 3:**
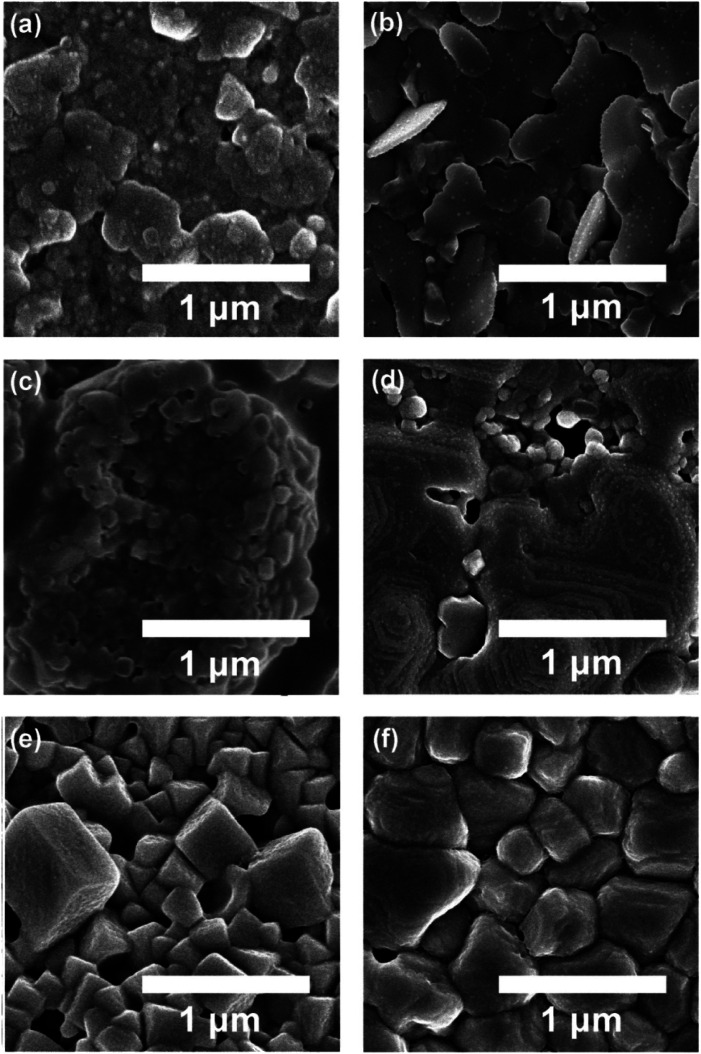
The SEM image of perovskite thin films deposited under different antisolvent conditions such as (a) ‘None’: without antisolvent, (b) CF, (c) CB, (d) DCB, (e) Tol, and (f) Et_2_O.

Fig. 3 shows the SEM image for perovskite thin films as a function of antisolvent species: (a) ‘None’, (b) CF, (c) CB, (d) DCB, (e) Tol, and (f) Et_2_O. Interestingly, as expected from the UV-vis absorption data, (f) Et_2_O-vapor and (e) Tol-vapor conditions display the most organized crystal structures with three-dimensional cubic or cuboid shapes. However, (a) ‘None’ and (b) CF vapor conditions show two-dimensional flake-like structures, whereas (c) CB and (d) DCB antisolvent conditions exhibit non-uniform morphologies. Finally, if the two conditions, (e) Tol and (f) Et_2_O are compared each other, the latter has a better morphology with uniformity and fewer pinholes than the former as shown in Fig. 3(e) and (f), suggesting that Et_2_O's properties such as *δ* = 7.4 (cal cm^−3^)^1/2^ and bp = 34.6 °C should be helpful to extract DMSO from the wet DMSO/perovskite film.

**Fig. 4:**
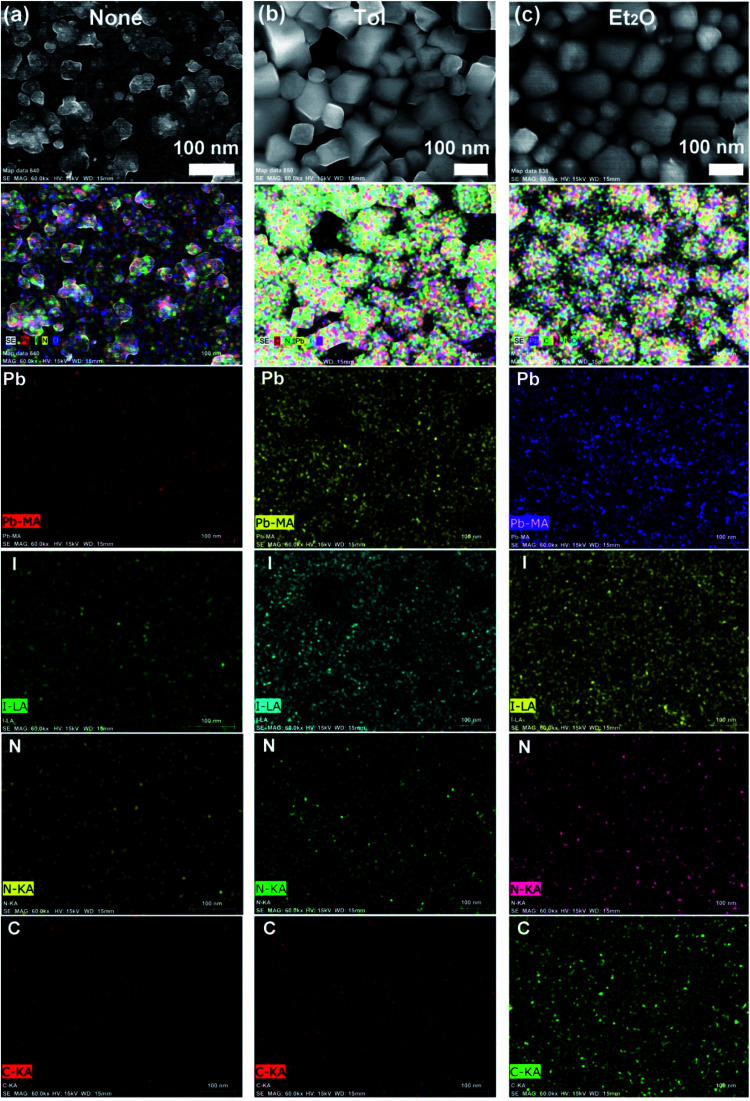
Elemental mapping images of the perovskite films according to each antisolvent condition: (a) ‘None’: without solvent–antisolvent vapors, (b) Tol, and (c) Et_2_O.

Fig. 4 shows the elemental mapping images of perovskite films for the three representative cases, (a) ‘None’, (b) Tol, and (c) Et_2_O. Here, the mapping data follows the morphologies of a sample according to the SEM images (Fig. 3). Accordingly, Et_2_O-treated perovskite film shows a uniform distribution of organic/inorganic elements, whereas Tol-treated one exhibits some voids/pinholes as shown in Fig. 4.

**Fig. 5:**
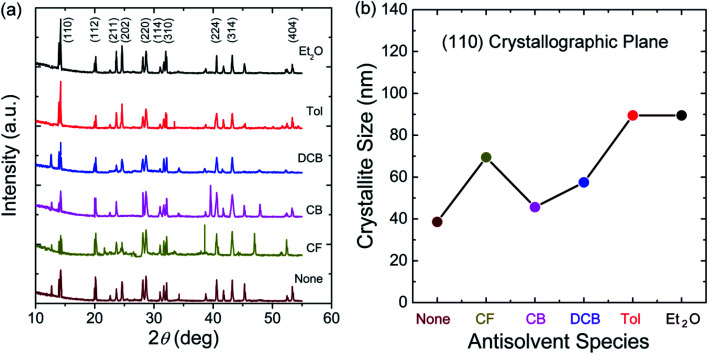
(a) XRD spectra of the perovskite thin films deposited under various antisolvent environments. (b) Crystallite size at the (110) crystallographic plane as a function of antisolvent species.

Fig. 5(a) shows XRD patterns for the perovskite film as a function of antisolvent species at room temperature. Importantly, CH_3_NH_3_PbI_3_ is a polymorphic material, exhibiting the crystal structures of orthorhombic at *T* < 162.2 K, tetragonal at 162.2 K < *T* < 327.4 K, and cubic at *T* > 327.4 K.^74^ Indeed, based on the data in Fig. 5(a), the calculated lattice parameters are *a* = *b* = 8.87 Å and *c* = 12.65 Å, confirming that perovskite has a tetragonal structure at ∼298 K according to the literature report.^75^ Interestingly, in Fig. 5(a), it is noticeable that ‘None/CF/CB/DCB’ conditions display unreacted PbI_2_ peak at 2*θ* ≈ 13°,^59^ whereas Et_2_O and Tol conditions do not exhibit such a peak from unreacted PbI_2_. This observation indicates that, in a modified sequential deposition process, PbI_2_ compounds would be reacted with MAI completely when DMSO-Tol or DMSO-Et_2_O was used as a solvent–antisolvent couple system. This is because Et_2_O [*δ* = 7.4 (cal cm^−3^)^1/2^ and bp = 35 °C] and Tol [*δ* = 8.9 (cal cm^−3^)^1/2^ and bp = 111 °C] are relatively nonpolar and volatile, allowing the wet DMSO/perovskite film to be dried fast (*i.e.*, the mixed DMSO-Tol or DMSO-Et_2_O molecules are quickly evaporated from the hygroscopic perovskite). This rapid crystallization results in a complete reaction between PbI_2_ and MAI. Furthermore, based on the most intense peak at (110) crystallographic planes in Fig. 5(a), the crystallite size of each perovskite film could be estimated. The result is displayed in Fig. 5(b). Importantly, the trend of crystallite size variation is in line with the UV-vis absorption data. However, one exception was observed in “CF’ condition which has volatile characteristics (bp = 61 °C). Recall the boiling point is in the order of 189 °C (DMSO) > 180 °C (DCB) > 131 °C (CB) > 111 °C (Tol) > 61 °C (CF) > 35 °C (Et_2_O). Table 2 shows the crystallite size of (110) crystallographic plane when *d*-spacing is 0.623 nm. Here the crystallite size (*t*) was calculated based on Scherrer's equation as follows,^76,77^2
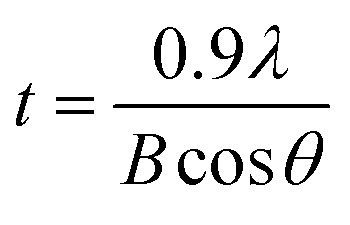
where *λ* (= 0.154 nm) is the wavelength of X-ray, and *B* is a full width at half maximum (FWHM) at diffraction angle, *θ*. Here, *d*-spacing was calculated based on the Bragg's law (*λ* = 2*d* sin *θ*).

**Table 2:** Crystallite size (*t*) of (110) crystallographic planes as a function of antisolvent species, when *θ* is 7.1°, X-ray wavelength (*λ*) is 0.154 nm, and *d*-spacing is 0.623 nm

	Antisolvent vapor when solvent vapor is DMSO					
	None	CF	CB	DCB	Tol	Et_2_O
*B* (rad)	0.00362	0.00201	0.00306	0.00243	0.00156	0.00156
*t* (nm)	38.6	69.5	45.6	57.5	89.5	89.5

**Fig. 6:**
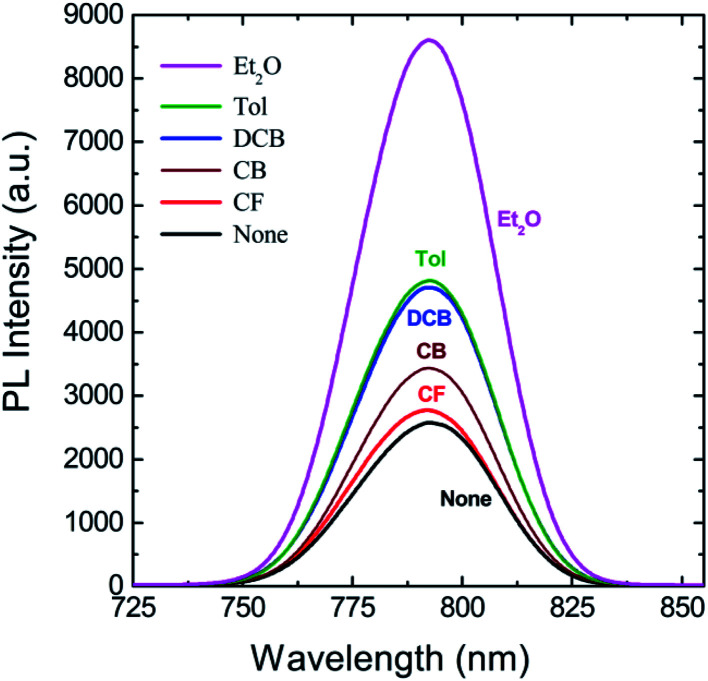
PL spectra for perovskite films as a function of antisolvent species. Note that here a perovskite film was directly deposited on the top of a glass slide (not PEDOT:PSS/ITO substrate) for the clarity of experiments.

Fig. 6 shows PL spectra for perovskite film as a function of antisolvent species, in which the peak was observed at 792.6 nm (‘None’), 792.0 nm (CF), 792.2 nm (CB), 792.5 nm (DCB), 792.6 nm (Tol), and 792.2 nm (Et_2_O), indicating the PL peak positions have no direct relationship with the optical bandgap (*E*_g_) shown in Fig. 2(b). However, the PL intensity has a direct correlation with the *E*_g_ in the UV-vis absorption data. For example, when *E*_g_ is 1.61 eV (the most red-shift sample), the PL intensity is highest, indicating that, when crystallite size is large in a well-organized morphology, the radiative recombination process is carried out abundantly, resulting in the highest intensity of PL. In other words, when morphologies have a lot of defects like in ‘None’ or ‘CF’ conditions, the probability of non-radiative recombination is increased, resulting in a weak intensity of PL as proved in Fig. 6.

**Fig. 7:**
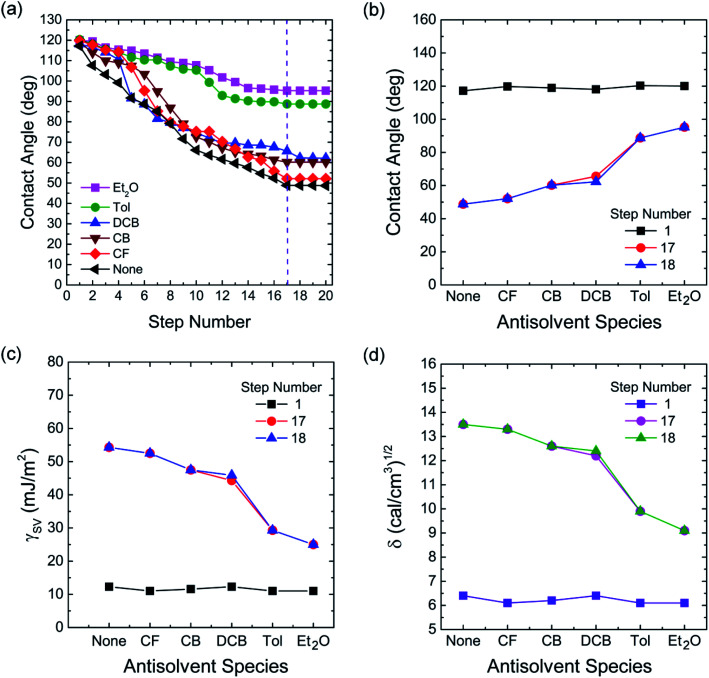
(a) Water contact angle (*θ*_*c*_) as a function of step number for each perovskite film made by different antisolvent vapor exposure. (b) Water contact angle, (c) surface energy, and (d) solubility parameter as a function of antisolvent species when the step numbers are 1, 17, and 18.

**Fig. 8:**
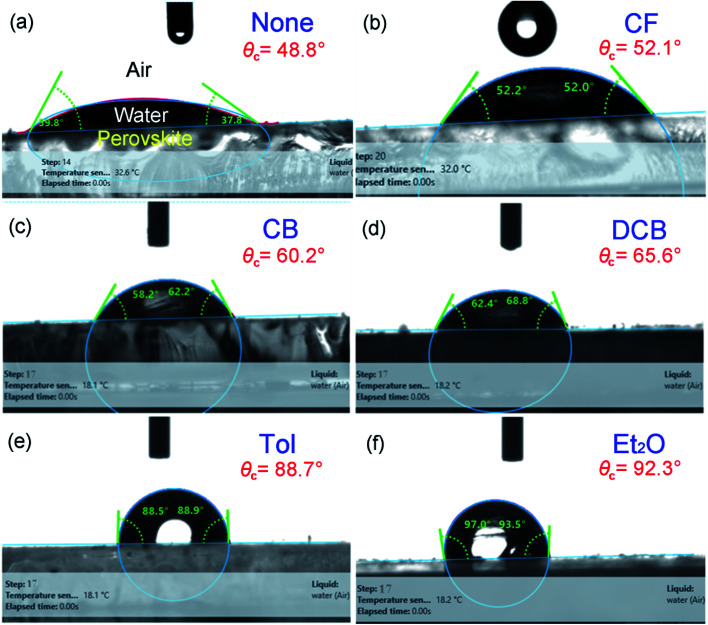
Examples of water contact angle (*θ*_*c*_) raw data at step number 17: (a) None, (b) CF, (c) CB, (d) DCB, (e) Tol, and (f) Et_2_O.

Finally, to understand the surface polarity of perovskite films depending on solvent–antisolvent vapor exposure, the water contact angle (*θ*_*c*_: here, subscript ‘*c*’ stands for contact angle) was measured (see Fig. 7 and 8). Here, it should be bear in mind that, when water is dropped on the surface of perovskite film, the nanoscale PbI_2_ film is known to be immediately formed at the interface between water and perovskite through degradation of CH_3_NH_3_PbI_3_.^63^ However, despite this PbI_2_ formation, the stability of perovskite film could be studied. This is because polycrystalline morphologies contain a lot of defects such as grain boundaries through which water molecules can be easily penetrated, resulting in the change of surface polarity of a film.

The raw contact-angle data at step number 17 is displayed in Fig. 8 as an example. Fig. 7(a) shows contact angle change as a function of step number in which each measurement was taken in time intervals of 40 ms over 30 s. In Fig. 7(a), the first striking observation is that, with increasing the step number, the contact angle decreased, indicating the polarity of a perovskite film was changed through the water-induced degradation effect. Note that in our previous work,^27^ the contact angle and surface energy for the pure PbI_2_ films (DMSO used as a processing solvent) were 130° and 6.3 mJ m^−2^, respectively. However, in this work, the perovskite film (from which PbI_2_ is formed, like a water/PbI_2_/CH_3_NH_3_PbI_3_ configuration) shows the water contact angle of about 120° and the average surface energy of ∼11.5 mJ m^−2^ (see Step 1 in Table 3). Hence, the water contact angle of a perovskite film should be affected by perovskite's degradation (PbI_2_), morphologies (including grain boundaries), and others.

**Table 3:** Water contact angle (°) and surface energy (*γ*_sv_) of organometal halide perovskite thin films at the step numbers of 1, 17, and 18

Antisolvent	Contact angle (°)			Surface energy (mJ m^−2^)		
	Step 1	Step 17	Step 18	Step 1	Step 17	Step 18
None	117.2	48.8	48.8	12.3	54.3	54.3
CF	119.8	52.1	52.1	11.0	52.5	52.5
CB	118.9	60.2	60.2	11.6	47.5	47.5
DCB	118.1	65.6	62.3	12.3	44.3	45.9
Tol	120.3	88.7	88.7	11.0	29.3	29.3
Et_2_O	120.0	95.3	95.3	11.0	25.0	25.0

The change of water contact angle with time was smaller for the cases of Et_2_O and Tol compared to the others, indicating that, when the perovskite materials were well crystallized (recall Fig. 3), the stability of films (*i.e.*, water-resistivity) should be significantly improved in humid conditions. The next observation is that at steps 17 and 18, the contact angle was saturated as shown in Fig. 7(b). In this study, it is noticeable that considering the golden triangle in solar cells (that is efficiency, stability, and cost),^78^ this stability-enhanced perovskite film should be important, providing a general insight for the necessity of a single crystal^70^ without any grain boundary as an ideal condition if there is a practical processibility.

## 4. Conclusion

The morphologies and surface properties of CH_3_NH_3_PbI_3_ thin films were studied by varying solvent–antisolvent vapor treatment conditions, for which the solvent was dimethyl sulfoxide (DMSO), and the antisolvents were chloroform (CF), chlorobenzene (CB), dichlorobenzene (DCB), toluene (Tol), and diethyl ether (Et_2_O). Major findings are as follows. First, according to UV-vis absorption data, the optical bandgap of perovskite films ranged from 1.61 eV (Et_2_O) to 1.63 eV (‘None’: without any solvent–antisolvent vapor treatment). Second, according to SEM images, when antisolvent was Et_2_O or Tol, the morphologies and crystal structures of perovskite films were improved. Third, when Et_2_O or Tol was used as an antisolvent, the precursor materials (PbI_2_ and CH_3_NH_3_I) were completely reacted (*i.e.*, without any PbI_2_ residue) according to the XRD patterns. Forth, according to PL emission data, when the crystallite size (*t* = 89.5 nm for both ‘Et_2_O’ and ‘Tol’ conditions) was large, the PL intensity was higher than those of the other conditions (DCB, CB, CF, and ‘None’). Fifth, by measuring the water contact angle as a function of antisolvent species, the surface energy (*γ*_sv_) of each perovskite film was estimated. Initially, the average *γ*_sv_ for all samples was 11.53 ± 0.64 mJ m^−2^. However, when the contact angle data were saturated at step number 17, the *γ*_sv_ values were different depending on the antisolvent condition: *γ*_sv_ = 25 mJ m^−2^ (Et_2_O), and *γ*_sv_ = 54.3 mJ m^−2^ (None), indicating that the high-quality films (exposed by Et_2_O) have more stability toward water compared to the others. Hence, the solvent–antisolvent vapor technique should be useful for the enhanced stability of perovskite layers if it is well utilized. Finally, our future works may include the device performances by extending the current study, leading to the processing-structure–property-performance relationship of perovskite solar cells.

## Conflicts of interest

The authors declare no competing financial interest.

## Supplementary Material
